# Effects of Mefenamic Acid in Pain Control during Loop Electrical Excision Procedure: A Prospective Double-Blind Randomized Control Trial

**DOI:** 10.31557/APJCP.2020.21.12.3633

**Published:** 2020-12

**Authors:** Poochit Dabpookhiew, Amornrat Temtanakitpaisan, Chumnan Kietpeerakool, Bandit Chumworathayi, Apiwat Aue-Aungkul, Fa-Ngam Chareonpol, Namphet Jumpathong

**Affiliations:** 1 *Department of Obstetrics & Gynecology, Faculty of Medicine, Khon Kaen University, Thailand. *; 2 *Department of Anesthesiology, Faculty of Medicine, Khon Kaen University, Thailand. *

**Keywords:** Loop electrosurgical excision procedure, mefenamic acid, pain control, preinvasive cervical cancer

## Abstract

**Objective:**

To investigate the effectiveness of pre-procedural oral mefenamic acid compared with placebo in women undergoing Loop Electrosurgical Excision Procedure (LEEP) with intracervical lidocaine injection.

**Study designs:**

A prospective double-blinded randomized control trial.

**Materials, Setting, Methods:**

Women undergoing LEEP for any indications were asked to participate in the study. The participants were randomly allocated into two groups. In group 1 (oral mefenamic acid), the participants were offered oral mefenamic acid (500 mg) for 30 minutes before procedures. In group 2 (placebo), the patients were given oral placebo (identical tablet) for 30 minutes before operation. All participants received immediate 10 mL of 2% lidocaine with 1:100,000 of epinephrine intracervical injection before undergoing the LEEP. All participants were excised in one piece of LEEP. No top-hat excision in this study. The patients graded their pain on a 10-cm visual analog scale (VAS) at different points during the procedure, including speculum insertion, at starting excision, and 30 minutes post excision. Primary outcomes revealed the difference of VAS during all steps of LEEP by generalized estimating equations procedure.

**Results:**

Sixty participants (30 in mefenamic group and 30 in placebo group) participated in this study. The study did not find differences of VAS during all steps of LEEP and analgesic drug requirement at 30 minutes after LEEP procedure. All patients reported no immediate complications and no intervention-related adverse events were observed.

**Conclusion:**

Using pre-procedural oral mefenamic acid before LEEP procedure was not associated with pain reduction during all steps of excision.

## Introduction

The Loop Electrosurgical Excision Procedure (LEEP) is widely used to treat high-grade cervical intraepithelial neoplasia (CIN2-3) to prevent invasive cervical diseases (Papalia et al., 2000). These procedures are usually performed in the outpatient setting, during which intracervical submucosal injection of lidocaine is generally performed to reduce pain. The other modalities of anesthesia for pain control during LEEP have been proposed in previous studies such as lidocaine spray, lidocaine gel, and oral analgesic. However, mild to moderate pain from patients who underwent LEEP are currently reported in speculum insertion, excision and post excision periods.To reduce mild to moderate pain, Mefenamic acid, a Nonsteroidal anti-inflammatory drug (NSAIDs), is widely used mostly in postoperative pain control (Shirvani et al., 2015). The actions of Mefenamic acid are achieved by blocking the effect of COX enzymes and decreasing prostaglandin products, which results in reduction of pain and inflammation (Cashman, 1996). Mefenamic acid is rapidly absorbed via oral administration and has half-life of action of two hours (Moll et al., 2011). Single dose of 500 mg oral mefenamic acid is commonly used before minor gynecologic procedure such as hysteroscope, fractional and curettage for intraoperative and postoperative pain control and complication avoidance (Buppasiri et al., 2005; Nagele et al., 1997). Due to insufficient data regarding whether adding pre-procedural oral analgesic medication can diminish pain in women undergoing the LEEP procedure, the aim of this study was to compare the effectiveness of pre-procedural additional mefenamic acid with the standard intracervical lidocaine injection for pain control in all steps of the procedure. 

## Materials and Methods

*Setting and population*

This double-blind, parallel-group, randomized controlled trial was conducted at Srinagarind Hospital between July 2019 and February 2020. Women who were diagnosed as abnormal cervical cancer and planned to undergo LEEP procedure at our center were asked to participate in this trial. As for inclusion criteria, the participants were to be 18-60 years of age and able to communicate well in Thai; meanwhile, those who had pregnancy, history of allergy to NSAID or lidocaine injection, peptic ulcer disease, coagulopathy, chronic kidney disease, and pelvic infection were excluded from the study. Participants were withdrawn from the analysis once found to have anaphylaxis signs and symptoms such as exacerbated respiratory symptoms (mucous hypersecretion, bronchoconstriction, nasal congestion, syncope) and mucocutaneous reactions (urticaria, angioedema, anaphylaxis) after receiving the pre-procedural oral medication. Ethical oversight was approved by the Khon Kaen University Ethics Committee for Human Research (HE611536). Written informed consent was obtained from all of the participants. This trial was performed and reported in compliance with the CONSORT statement (Schulz et al., 2010) and was registered with the Thai Clinical Trial Register (TCTR20190307001). 

*Sample size calculation*

The mean ± SD of pain score (VAS) from all steps of LEEP, including speculum insertion, excision, and 30-minute post excision were the primary outcome. Sample size in this study was calculated for analysis of generalized estimating equations based on a study by Vanichtantikul et al., 2013). We hypothesized that a combination of pre-procedural oral mefenamic acid with intracervical lidocaine injection before LEEP (group1) would achieve a higher score of pain reduction in all stages of LEEP procedure compared to that of group receiving placebo (group2). Based on a power of 80%, a type I error of 0.05 and the sample size needed for testing our primary outcome was 60 women, 30 of whom were assigned in each group. 

*Randomization and intervention*

The participants who met the inclusion criteria were randomly allocated to one of the comparison groups using computer generated block randomization of varying block size. The assigned adjuvant treatment was noted on cards, which were sealed in secure opaque envelopes. The envelopes were then numbered in sequence, kept and opened by an independent researcher in an office outside the hospital. All LEEP procedures were performed by five gynecologic oncologists. The surgeon, participants and investigator involved in this study were blinded to the intervention allocated. Before performing LEEP procedures, participants having been allocated to the intervention group (group1) were given oral mefenamic acid (500 mg) at 30 minutes before LEEP procedures. Meanwhile, the participants having been allocated to the control group (group2) received oral placebo at 30 minutes before LEEP procedure. Oral placebo was prepared with the appearance similar to that of Mefenamic acid. All participants received the same operative excision procedure with local anesthesia under 10 mL of 2% lidocaine with 1:100,000 of epinephrine intracervical injection. All participants were excised in one piece of LEEP. No top-hat excision in this study. To minimize the effects of other variables, oral administration of others analgesic drugs or injected analgesic drugs before excision were not allowed.

*Outcomes and measurements*

According to the primary outcomes, VAS was different in all steps of LEEP procedures between two groups. Secondary outcomes were additional analgesic required at 30 minutes after LEEP procedure and adverse events. Pain scores were assessed by 10-cm visual analogue scale from 0 (no pain) to 10 (a maximum level of experienced pain). An investigator asked all individual participants to point their pain score based on a standard 10-cm visual analogue scale. Pain score assessments were recorded before speculum examination, at starting excision and at 30 minutes after LEEP procedure. The participants were instructed to inform nurses or investigators if analgesic drug was required at 30 minutes after LEEP procedure and if single dose of 500 mg oral paracetamol was given. Safety assessments were then recorded, including adverse events and immediate complications within 30 minutes after LEEP.

*Statistical analyses*

Statistical analyses were performed using Stata 10 (Stata Corporation, College Station, Texas). Descriptive statistics were used to report participants’ baseline characteristics. Student t-test was used to compare continuous variables. In addition, the Chi-square or Fisher-exact test were employed to compare categorical variables. Differences between the comparison groups were measured, including mean difference (MD) or relative risk (RR) with their associated 95% confidence interval (CI). Statistical analysis was conducted using generalized estimating equations. P-value < 0.05 was considered to be clinically significant. All analyses were carried out based on the intention-to-treat principle.

## Results

**Figure 1:**
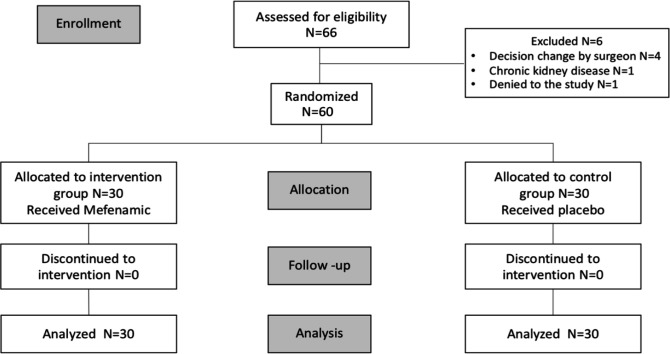
Flow Diagram of Trial Recruitment and Follow-up Evaluation

**Figure 2:**
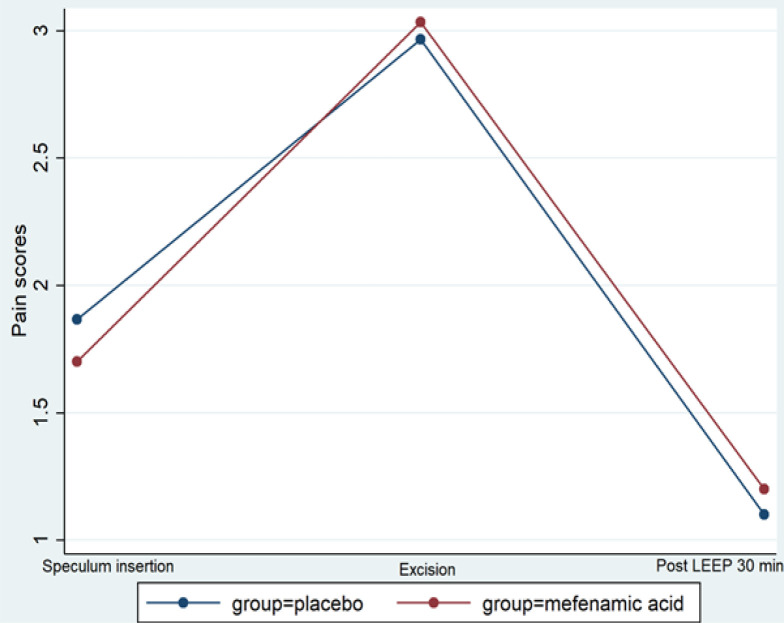
Difference in Pain Scores between Intervention and Control Groups at Each Time Point

Of the study population, 66 women were assessed for eligibility, 60 of whom met the inclusion criteria and were randomly allocated to either the mefenamic group (n = 30) or the control group (N = 30). Figure 1 shows the CONSORT flow diagram of this study. Six participants were excluded from this study. Four patients were subjected to change by the doctor’s decision; one patient denied participating in the study; and one was found to have an underlying disease - chronic kidney disease, which is vulnerable to NSAIDs. No participants withdrew after the assignment of the intervention. Demographic data of patients in both arms were similar (Table 1). The mean age ±SD was 44.5± 10.7 years. A total of 85% of patients were multiparous, 73% of whom were identified as in premenopausal status and 71.1% identified no underlying disease. High-grade squamous intraepithelial lesion was found to be the most common abnormal cervical cytology in both groups. The loop diameter we used most in our study (71.7%) were 1 cm and its size were similar in both groups (Table 1). We observed significant difference of final pathology between two groups (60% of HSIL in intervention group and 33.3% of HSIL in control group; p=0.038). However, the differences of final pathology did not affect primary or secondary outcomes. The severity of pain was assessed by means of a VAS at each time point. The mean VAS value during excision was 3.03 ± 2.68 for the group treated with Mefenamic acid and 2.97± 2.34 for the placebo group. Mean values of VAS were 1.20 ± 2.06 for the group treated with Mefenamic acid and 1.1± 1.35 for the placebo group at 30 minutes after LEEP procedure. The mean difference between two groups (group 2 versus group 1) in all steps of LEEP procedures was -0.17 (95%CI; -1.10 to 0.77) (Figure 2). 

According to the Table 2, administering pre-procedural Mefenamic acid 30 minutes before LEEP procedure did not result in a significantly reduced pain score during excision (MD 0.23; 95% CI -0.86, 1.33) and at 30 minutes post LEEP procedure (MD 0.27; 95% CI -0.89, 1.42). In addition, no difference was observed when using analgesic drug at 30 minutes after LEEP procedure (RR 0.67; 95% CI 2.27, 1.64, p=0.371). 

When subgroup analysis was applied, the size of the loop diameter did not affect the pain score in both groups (Table 3). Moreover, parity, menopausal status, cervical cytological and final histological type were not associated with the pain score during excision and post LEEP 30 minutes. All participants reported no observed immediate complications and no intervention-related adverse events were exhibited.

**Table 1:** Baseline Characteristics of the Participants

Characteristic	Total (n=60)		Drug (n=30)		Control (n=30)		P value
Age (year); mean ±SD	44.5	±10.7	43.2	±9.8	45	±11.5	0.3332
Parity*							0.472
Nulliparus	9	(15)	3	(10)	6	(20)	
Multiparus	51	(85)	27	(90)	24	(80)	
Menopausal status							0.243
Premenopause	44	(73.3)	24	(80)	20	(66.7)	
Postmenopause	16	(26.7)	6	(20)	10	(33.3)	
Underlying disease							0.39
No	43	(71.7)	20	(66.7)	23	(76.7)	
Yes	17	(28.3)	10	(33.3)	7	(23.3)	
Cervical cytology							0.602
≥HSIL	34	(56.7)	18	(60)	16	(53.3)	
≤LSIL	26	(43.3)	12	(40)	14	(46.7)	
Loop diameter							0.39
1 cm	43	(71.7)	20	(66.7)	23	(76.7)	
>1 cm	17	(28.3)	10	(33.3)	7	(23.3)	

Data are expressed as n (%); *, Fisher's exact test

**Table 2:** Analytic Pain Score in Each Stage of Procedure between Two Groups

Parameters	Mean difference	95% CI	P value
Group			
G2 VS G1	-0.17	-1.10 to 0.77	0.727
Time			
T_2_ VS T_1_	1.1	0.33 to 1.86	0.005
T_3_ VS T_1_	-0.77	-1.47 to -0.07	0.032
Group x time			
(G_2_T_2_ VS T_1_) VS (G_1_T_2_ VS T_1_)	0.23	-0.86 to 1.33	0.677
(G_2_T_3 _VS T_1_) VS (G_1_T_3_ VS T_1_)	0.27	-0.89 to 1.42	0.652

GEE, generalized estimating equations; G_1_, placebo group; G_2_, drug group; T_1_, Speculum insertion; T_2_, Excision; T_3_, Post LEEP 30 min

**Table 3:** Estimate Difference in Pain Scores between Intervention and Control Groups (Subgroup Analysis by Loop Diameter)

Parameters	1 cm (n=43)			>1 cm (n=17)		
Estimate	95% CI	P value	Estimate	95% CI	P value
Group						
G_2_ VS G_1_	-0.19	-1.37 to 0.99	0.757	0.16	-1.12 to 1.44	0.810
Time						
T_2_ VS T_1_	0.78	-0.14 to 1.70	0.096	2.14	1.19 to 3.09	<0.001
T_3 _VS T_1_	-0.87	-1.72 to -0.02	0.044	-0.43	-1.57 to 0.72	0.463
Group x time						
(G_2_T_2_ VS T_1_) VS (G_1_T_2_ VS T_1_)	0.32	-1.00 to 1.64	0.638	-0.34	-2.03 to 1.34	0.690
(G_2_T_3_ VS T_1_) VS (G_1_T_3_ VS T_1_)	0.12	-1.28 to 1.52	0.868	0.43	-1.55 to 2.41	0.671

GEE, generalized estimating equations; G_1_, placebo group; G_2_, drug group; T_1_, Speculum insertion; T_2_, Excision; T_3_, Post LEEP 30 min

## Discussion

The results of this study demonstrated that the administration of pre-procedural Mefenamic acid 30 minutes before LEEP procedure did not result in statistically significant relief pain score in women who underwent LEEP procedures compared with placebo. Moreover, no differences in analgesic drug required at 30 minutes after LEEP procedure were observed. Furthermore, pre-procedural Mefenamic acid before LEEP was considered safe as no intervention-related adverse events took place. Preinvasive cervical cancer especially high-grade lesion are usually treated with excision procedures; thus, most tertiary hospitals performed LEEP excision in the outpatient setting (colposcopy clinic). Moreover, several guidelines recommended the analgesic prior to excisional procedures. Indeed, multiple anesthetic modalities for pain control during LEEP are available such as lidocaine gel, lidocaine spray, intracervical lidocaine injection, paracervical block (Vanichtantikul et al., 2013; Harper et al., 1994; Limwatanapan et al., 2018). Premedical with NSAID can be used adjuvant with anesthetic block for additional pain control (Schulz et al.,2010; Limwatanapan et al., 2018; Gajjar et al., 2016; Lipscomb et al., 1995) . A previous study reported that premedication with oral Mefenamic acid 500 mg significantly reduced pain in 30 and 60 minutes after hysteroscopy (Nagele et al., 1997). However, using oral Mefenamic acid 500 mg did not show the significant difference of pain score in fractional and curettage compared with paracervical block (Buppasiri et al., 2005). 

Two small RCTs was used to evaluate oral naproxen before carbodioxide laser vaporization for preinvasive cervical cancer. Al-Kurdi et al., 1985 reported that premedication oral NSAIDs (Naproxen sodium) used in carbondioxide laser treatment of uterine cervix has minimal effect on the pain and causes discomfort during laser treatment. The likely explanation for the failure of naproxen sodium to relieve the pain during laser application may be that transmitting nerves are stimulated directly during applying the laser. To illustrate, naproxen sodium is not a centrally acting analgesic and would not be expected to reduce this sort of pain. However, naproxen sodium appears to have some advantages over placebo in the period following treatment when a response to tissue damage takes place. Therefore, if being routinely given naproxen sodium before laser treatment, patients may be prevented from distress after treatment. Frega et al., (1994) evaluated pain score during carbon dioxide laser vaporization for cervical intraepithelial neoplasia (CIN) among 3 groups; naproxen, placebo and no treatment. It may be argued from the study that analgesia before laser surgery for CIN is not required. In the present study, we reported pre-procedural Mefenamic acid before LEEP and we also found that it was not necessary either to relieve pain in women undergoing LEEP. A Cochrane review conducted by Gajjar et al., (2016) revealed that there was no difference in pain score in women receiving oral analgesics compared with placebo or no treatment during colposcopy treatment and in the postoperative period (MD -3.51; 95% CI -10.03 to 3.01; 129 women). The authors suggested that the evidence to be of a low to moderate quality and required further high quality, adequately powered trials to provide the data necessary to estimate the efficacy of oral analgesics, the optimal route of administration and dose of local anesthetics. Similarly, our study confirms that pre-procedural oral Mefenamic acid before LEEP is unlikely to significantly reduce pain during LEEP excision and post LEEP at 30 minutes. Al-Kurdi et al., 1985 reported that patients suffered from some pain during laser treatment particularly from dysmenorrhea, dental drilling and parturition. However, our study indicated that the factors such as the size of loop diameter, parity, menopausal status, cervical cytological and final histological type are not associated with pain score during excision and post LEEP 30 minutes by subgroup analysis. Moreover, in this study, a total of 70% of participants were performed LEEP by using 1 cm of loop diameter if using larger loop, it possible to be showed the different result.

The strength of this study is that it was a prospective double-blinded randomized control trial, in which we used placebo prepared in similar appearance as Mefenamic acid. Another strength of this current study was that no participants dropped out from the study. The LEEP excision was performed in the standard technique by the same level of operators. In addition, as for postoperative pain evaluation, we applied analgesic using pain scores in order to address the bias of individual experience pain. Furthermore, this study is the first trial that evaluated the difference of pain scores in each time points and both groups were evaluated using generalized estimating equations. 

In conclusion, using pre-procedural oral Mefenamic acid before LEEP procedure is unlikely required. Further larger investigation with different approach in higher dose of Mefenamic acid and other pre-procedural oral analgesic administration would be explored to optimize pain during LEEP procedures. 
